# Birth preparedness complication readiness and determinants among pregnant women: a community-based survey from Ethiopia

**DOI:** 10.1186/s12884-020-03297-w

**Published:** 2020-10-19

**Authors:** Teshale Abosie Ananche, Legesse Tadesse Wodajo

**Affiliations:** 1 Center of International Program ICAP, Addis Ababa, Ethiopia; 2 Semera Regional Office, Semera, Afar Regional State Ethiopia; 3 Department of Public Health, College of Health Science, Arsi University, Assela, Ethiopia

**Keywords:** Delivery, Birth preparedness, Complication readiness, Afar region, Ethiopia

## Abstract

**Background:**

Maternal death related to obstetric complications remains a great challenge in developing countries. Since these complications are not consistently predictable, it is important to plan different preventive approaches to overcome them when. As the information on birth preparedness, complication readiness, and predictors were limited in the study area, we conducted this study.

**Methods:**

A Cross-sectional study involving 396 pregnant women was conducted from 1st April to 1st May 2018. Data were collected using a pre-tested structured questionnaire. Descriptive, binary and multiple logistic regression analyses were conducted in SPSS for windows version 20. *P* values < 0.05 were considered significant.

**Results:**

Of 361 women interviewed (91% response rate), birth preparedness and complication readiness were present in 24.10% (87/361) of women. Maternal factors, age 18–19 (AOR = 0.18; 95% CI (0.04,0.94)), 20–34 (AOR = 0.40; 95% CI (0.20,0.78)), education, not able to read/write (AOR = 0.36;95% CI (0.15,0.85),read/write (AOR = 0.41;95% CI (0.19,0.89)), Muslim religion (AOR = 0.40; 95% CI (0.18,0.85)) income ETB, < 1000 (AOR = 0.21; 95% CI (0.07,0.67)),1000–2000, (AOR = 0.38; 95% CI (0.19,0.76)), and the mothers’ knowledge on key danger signs of postpartum (AOR = 0.48; 95% CI (0.26,0.90)) were independent predictors of birth preparedness and complication readiness.

**Conclusions:**

Educational status, age, religion, family income, and knowledge of obstetric danger signs were significantly associated with birth preparedness and complication readiness. The Government and other health sector partners should work to improve women’s education, income, and focus on young age groups on pregnancy danger signs.

## Backgrounds

Maternal mortality is an unsettled public health agenda [1]. A large proportion of mothers living in developing countries remain in need of improved living conditions [2]. As of 2015, the two regions with the highest Maternal Mortality Rate (MMR) were Sub-Saharan Africa and Oceania [3]. In Ethiopia, MMR remains high (412 deaths per 100,000 live births according to 2016 Ethiopian Demographic and Health Survey (EDHS)), with more than 73% of mothers in delivering at home without any help from skilled attendants [4].

Birth preparedness and complication readiness (BP/CR) is a method aimed at promoting timely access to skilled maternal and neonatal services. This approach has its tools and indicators for monitoring maternal as well as neonatal health [5]. BP/CR helps pregnant women and their families to actively prepare and make informed decisions related to delivery. In addition, the majority of relatives and the community at large also lack information on these signs. During the occurrence of complications, the unprepared woman and relatives spend an excessive amount of time to tackle the danger. For example, a series of issues like getting organized, collecting money, and finding transportation to the appropriate Hospital facility are all the sources of delay. In addition, further delays could also occur in a health-care facility for identifying a compatible blood donor in case of hemorrhage or obtaining written consent before the actual help [6–8]. According to studies conducted in some parts of Ethiopia, only 16.5 to 22% of pregnant women were found to be prepared for birth and complications [9–11] which is similar to other developing nations like Kenya and India [12, 13].

Despite all the benefits of preparation for childbirth and readiness for unseen complications, limited information is available on this issue in the Afar region, Ethiopia. Therefore, this study explored the degree of preparation and readiness for delivery related issues in the study area. As a secondary objective this study also identified factors associated with BP/CR.

## Methods

### Study setting

A community based cross-sectional study was conducted from April 1st to May 1st, 2018 in Amibara Woreda of Afar Region, Ethiopia. Amibara is one of 34 Woredas from the Afar Regional State and is located 280 km away from Addis Ababa, (the capital city of Ethiopia). It is found in Zone-3 (*Geberesu Zone*) administrative part that covers an area of 3994.00 Square Kilometre. Amibara Woreda has sixteen rural Kebeles and three Urban Kebeles [4].

According to the Central Statistical Agency (CSA) of Ethiopia, the estimated population of Amibara Woreda was 81,811in 2016 [4]. There is one hospital, four health centres, and eighteen health posts in the woreda. Most of the Afar population are pastoralists who rely on livestock production. The languages spoken are- Afarigna and Amharic. Only 7.7% of the urban population are literate with an overall (urban and rural) literacy rate of less than 3%.. The main religions in the area are either Islamic or Christianity whereas the predominant ethnic groups are Afar, Amhara, and Argoba [14].

### Study participants

**A**ll pregnant women residing in the study woreda were the source population whereas 396 pregnant women living in selected kebeles were included in our study population.

The sample size was calculated based on a single population proportion formula. The total population of reproductive age women in Amibara woreda was about 35,056, of which 904 were expected to be pregnant. From a previous study result in the Bale zone, it was assumed 29.9% of pregnant women were well prepared for delivery and complications [7]. Then take a 95% confidence level and 5% margin of error (d), the sample was 322 women. After a design effect of 2, it gives 644 and 64 participants were added for a non-response rate to make 708. Finally, the sample size becomes 396.

The sampling procedure used was a multistage sampling method. Amibara woreda was chosen by lottery method (from the 34 woredas in Afar region), and its 19 kebeles were stratified into three urban and 16 rural.

After the total number of pregnant women in Amibara woreda was identified from a Health Extension Workers’ registration book, the proportions of the pregnant women to be taken from the four selected kebeles were calculated. Then, in order to take one participant from one household, a household sampling unit to be drawn from each kebele was identified. The study units (Households) taken from each selected kebele were in proportion to the population size, and every unit was approached by the Systematic Random Sampling (SRS) method. Meaning, after the first household, to begin with, was selected randomly, a sampling fraction between the sample size and population size was used to recruit further participants. In case there were two or more eligible women in one household, only one was selected randomly. Absentees during three home visits made by interviewers were labeled as a non-response. Pregnant women who were seriously sick were excluded from the study. All eligible women in the household were then interviewed.

### Study variables

The dependent variable was BP/CR whereas the independent variables were grouped into three categories. These were socio-demographic factors in the first category obstetric factors in the second category and factors related to the awareness of a pregnant woman about the danger signs of pregnancy and delivery in a third category.

### Operational definitions

Birth preparedness (BP/CR) is a preparation that is planned well before delivery. A woman was considered to be well prepared for birth if she has identified a place of delivery, skilled birth attendant, saved money, and identified the mode of transport ahead of delivery. A participant who fulfilled at least three of the above criteria was considered well-prepared. Complication readiness was defined as as-an awareness of pregnancy and delivery related health problems and was considered to be present when a woman made all the necessary plans for emergency situations. For example, the arrangement of (funds, transport, blood donor, and designated decision maker) in case of hemorrhage and loss of consciousness.

A skilled care provider/attendant is a professional caregiver who has the knowledge and skills to manage labor, childbirth, and the postpartum period. She must be able to recognize complications and refer to the woman or the newborn to a higher level of care if the complications require interventions beyond the skilled attendants’ competence.

The postpartum period was defined as a time starting from the delivery of the placenta up to 6 weeks. A newborn/neonatal period refers to the first 7 days of the newborn’s life.

### Measurement tools and definitions

A pre-tested interview questionnaire was used for data collection. The questionnaire was adapted from a JHPIEGO tool for monitoring of birth preparedness and complication readiness and indicators for maternal and newborn health [5]. Data were collected on sociodemographic variables like age, marital status, residence (urban versus rural), ethnicity, religion, education, occupation, and average monthly family income.

The questionnaire also included inquiries on health problems related to the current pregnancy, danger signs of pregnancy, and awareness on issues of delivery and postnatal period which require referral.

A woman was considered to be knowledgeable on key danger signs of pregnancy, if she mentioned at least three of the eight key danger signs for pregnancy (vaginal bleeding, swollen hands/face, and blurred vision) spontaneously [9, 15]. Likewise, a woman was considered as knowledgeable on the key danger signs of labor/childbirth, if she spontaneously mentioned at least three of the five major danger signs for labor/childbirth (severe vaginal bleeding, prolonged labor (> 12 h), convulsion and retained (placenta) [9, 15] or a-.

Mention of at least two of the four key danger signs of a postpartum period (severe vaginal bleeding, foul-smelling vaginal discharge, and high fever) spontaneously [9, 15]. The women were also asked about their antenatal care visits and preferred place of delivery.

### Data processing and analysis

Data quality was maintained by proper recruitment, training, and supervision of data collectors. Data were checked for completeness, accuracy, and consistency by the principal investigator, and corrective measures were taken during data collection. The data was then entered into EPI-Info version 7.2.1.0 software and subsequently exported to SPSS version 20 statistical programs for analysis. The data were cleaned and descriptive statistics (using frequencies) were computed for each variable. Besides, bivariate and multiple regression analyses were carried out to identify independent predictors. During the analyses 95% confidence interval (CI) for odds ratio (OR) and *P*-value < 0.05 were used in declaring statistical significance.

### Ethics approval

The study was commenced after obtaining ethical approval from the Ethical Review Committee of Adama General Hospital and Medical College. Informed consent was obtained from all participants before the interview.

## Results

### Sociodemographic characteristics

During this study among 396 pregnant women identified 361 completed the interview (making a response rate of 91.00%). The mean age of the respondents was 29 ± 3 years.

Of the respondents 315 (87.26%) were currently in marital union, 260(72.02%) were urban and the remaining rural, 146(40.44%) were from Afar ethnic group. Muslim religion followers were 166(45.98%). Education status showed 147 (40.72%) of respondents cannot read and write while 84(23.26%) were able to write and read, 151 (41.83%) were housewives (Table 1).

**Table 1:** Sociodemographic characteristics of pregnant women involved in Amibara Woreda, Afar Region, Ethiopia, 2018

Variable	Variable category	Frequency	
		N	Percent (*N* = 361)
**Residence**			
	Urban	260	72.02
	Rural	101	27.98
**Age category**	18–19	23	6.37
	20–34	270	74.79
	35–49	68	18.84
**Marital Status**	Single	23	6.37
	Married/in union	315	87.26
	Divorced	10	2.77
	Widowed	13	3.60
**Religion**	Orthodox	118	32.69
	Catholic	13	3.60
	Protestant	64	17.73
	Muslim	166	45.98
**Ethnic group**	Afar	146	40.44
	Amhara	77	21.33
	Oromo	43	11.91
	Tigere	16	4.43
	SNNP^a^	79	21.88
**Occupation of the woman**	Cattle production	60	16.62
	House wife	151	41.83
	Government employee	87	24.10
	Private employee	25	6.93
	Private business	25	6.93
	Jobless	13	3.60
**Educational status of the mother**	Not read and write	147	40.72
	Read and write	84	23.26
	Primary	51	14.13
	Secondary and above	79	21.88
**Monthly income of the household**	< 1000 ETB	48	13.30
	1000–2000 ETB	117	32.41
	> 2000 ETB	196	54.29
**Family size**	< 4	253	70.08
	> 4	108	29.92
**Occupation of the husband**	Nomad	86	23.82
	Farmer	14	3.88
	Government employee	185	51.25
	Private employee	38	10.53
	Private business	38	10.53

^a^ *SNNP* South Nations, Nationalities and Peoples

### Obstetric characteristics of the participants

About 316 (87.53%) of respondents attended antenatal care in their current pregnancy. Concerning gestational age of the participants 249(68.98%) were less than three complete months while remaining were three or more. As of the parity of the participants, 82(22.71%), had zero, 161(44.60%) less than three and the remained more than three. Stillbirth history of the study women revealed 48(17.33%) had it. Two hundred ninety three (92.72%) accessed advice from health professionals while 16(5.06%) from Health extension Workers (Table 2).

**Table 2:** Obstetric characteristics of pregnant women in Amibara Woreda, Afar Region, Ethiopia, 2018 (*N* = 361)

Variable	Variable category	Frequency	
		N	%
**Gestational age of the current pregnancy**	< 3 Months	156	43.21
	3–6 Months	55	15.24
	> 7 Months	150	41.55
**Gravidity**	Gravida < 3	249	68.98
	Gravida > = 3	112	31.02
**ANC visits in current pregnancy**	Yes	316	87.53
	No	45	12.47
**No. ANC visits(316)**	1 time	76	24.05
	2 times	126	39.87
	3 times	79	21.88
	4 times	35	11.08
**Service providers of current ANC (*N*** **= 316)**	Health professional^a^	293	92.72
	Health Extension Worker	16	5.06
	Don’t know	7	2.22
**Parity**	Para 0	82	22.71
	Para 1–3	161	44.60
	Para > = 3	118	32.69
**Stillbirth history(*N*** **= 279)**	Yes	48	17.20
	No	231	82.80
**Stillbirth (numbers of times)(*N*** **= 48)**	1 time	44	91.67
	2 times	4	8.33

^a^ Health professional (clinicians, nurses, midwives …)

### Knowledge of danger signs during pregnancy, labor/childbirth, and postpartum time

Among 361 pregnant women participated in this study, 277 (76.73%) spontaneously mentioned at least one key danger sign. From two hundred seventy seven respondents, 210 (58.17%) mentioned vaginal bleeding. Accelerated/reduced fetal movement 16 (4.43%), water break without labor in 48 h (13.30%) and blurred vision 21 (5.82%) were spontaneously mentioned (Table 3).

**Table 3:** Birth Preparedness and Complication Readiness among pregnant women in Amibara Woreda, Afar Region, Ethiopia, 2018. (N = 361)

Birth Preparedness and Complication readiness		Frequency	
		Number	Percent
**Prepared well**	Yes	87	24.10
	No	274	75.90
**Exposure to BPCR Information**	Yes	319	88.37
	No	42	11.63
**Source of information about BPCR (*n*** **= 319, multi-response)**	Health Professional	217	60.11
	Health Extension Worker	44	12.19
	Trained Traditional Birth Attendant	9	2.49
	Traditional Birth Attendant	24	6.65
	Media	59	16.34
	Mothers	94	26.04
**Prepare essential item or clean delivery**	Yes	230	63.71
	No	131	36.29
**Identify health facility for childbirth**	Yes	296	81.99
	No	65	18.01
**Arranged transport**	Yes	60	16.62
	No	301	83.38
**Saved money**	Yes	159	44.04
	No	202	55.96
**Arranged blood donor**	Yes	5	1.39
	No	356	98.61
**Identify skilled birth attendant**	Yes	150	41.55
	No	211	58.45

### Birth preparedness and complication readiness

Proportion of mothers with BP/CR among the study groups in Amibara District of Afar Region in Ethiopia was 24.10%. Among those respondent who knew about BP/CR, 296 (81.99%) spontaneously identified and mentioned place of delivery, 230 (63.71%) prepared essential items for clean delivery and postpartum period, 150 (41.55%) identify skill provider, arranged blood donor 5 (1.39) followed by saving money; 159 (44.04%) (Table 4).

**Table 4:** Characteristics of knowledge of danger signs of pregnancy among participants, Amibara Woreda, Afar Region, Ethiopia, 2018. (N = 361)

Variables	Response Yes Or No	Awareness					
		Pregnancy		Labor/child birth		Postpartum	
		n	%	n	%	n	%
Knowledge of Obstetric Danger signs	Yes	277	76.73	282	78.12	196	54.29
	No	84	23.27	79	21.88	165	45.71
Vaginal bleeding	Yes	210	58.17				
	No	151	41.83				
Blurred vision	Yes	21	5.82				
	No	340	94.18				
Swollen hand/face	Yes	139	38.50				
	No	222	61.50				
Convulsion	Yes	31	8.59				
	No	330	91.41				
Loss of consciousness	Yes	38	8.53	52	14.40	65	18.01
	No	323	90.47	309	85.60	296	81.99
Severe abdominal pain	Yes	81	22.44				
	No	280	77.56				
Amniotic fluid leak before labor start	Yes	48	13.30				
	No	313	86.70				
Accelerated/reduced fetal movement	Yes	16	4.43				
	No	345	95.57				
Severe vaginal bleeding	Yes			177	49.03	144	39.89
	No			184	50.97	217	60.11
Placenta not delivered in 30 min	Yes			164	45.43		
	No			197	54.57		
Labor last > 12 h	Yes			173	47.92		
	No			188	52.08		
Having high grade fever	yes					41	11.36
	No					320	88.64
Foul smelling vaginal discharge	yes					47	13.02
	No					314	86.98

### Factors associated with birth preparedness and complication readiness

Birth preparedness and complication readiness was less likely made in younger age of 18–19 and 20 to 34 years old women than old age of 35 to 49, (AOR = 0.18, 95% CI (0.04, 0.94)) and (AOR = 0.40, 95% CI, (0.20,0.78)) respectively. Women who have no knowledge of post-partum related complication were found less likely prepared for B/CR (AOR = 0.48, 95% CI (0.26, 0.90)) (Table 5).

**Table 5:** Factors affecting BP/CR, Amibara Woreda, Afar Region, Ethiopia, 2018. (N = 361)

Variable	Category	well prepared		COR 95% (CI)	AOR 95% (CI)
		Yes N (%)	No N (%)		
Age	18–19	2 (8.70)	21 (91.30)	**0.19 (0.04,0.87)**	0.18 (0.04,0.94)*
	20–34	62 (22.96)	208 (77.04)	0.58 (0.33,1.04)	0.40 (0.20,0.78)*
	35–49	23 (33.80)	45 (66.20)	1.00	1.00
Religion	Catholic	5 (38.46)	8 (61.54)	1.11 (0.33,3.81)	0.42 (0.11,1.67)
	Muslim	26 (15.66)	140 (84.34)	**0.33 (0.17,0.64)**	0.40 (0.18,0.85)*
	Orthodox	33 (27.97)	85 (72.03)	0.69 (0.36,1.33)	0.48 (0.23,1.01)
	Protestant	23 (35.94)	41 (64.06)	1.00	1.00
Maternal Education	Not read and write	19 (12.92)	128 (87.07)	**0.19 (0.10,0.36)**	0.36 (0.15,0.85)*
	Primary	15 (29.41)	36 (70.59)	0.52 (0.25,1.11)	0.74 (0.32,1.71)
	Read/ write	18 (21.43)	66 (78.57)	**0.34 (0.17,0.68)**	0.41 (0.19,0.89)*
	Secondary and above	35 (44.30)	44 (55.70)	1.00	1.00
Income (ETB)	< 1000	4 (8.33)	44 (91.67)	**0.18 (0.06,0.51)**	0.21 (0.07,0.67)*
	1000–2000	16 (15.84)	101 (84.16)	**0.31 (0.17,0.56)**	0.38 (0.19,0.76)*
	> 2000	67 (34.18)	129 (65,82)	1.00	1.00
Knowledge of pregnancy related cxn.	No	15 (17.86)	69 (82.14)	0.61 (0.33,1.16)	1.12 (0.45,2.78)
	Yes	72 (25.99)	205 (74.01)	1.00	1.00
Knowledge Labor related cxn.	No	14 (17.72)	65 (82.28)	0.62 (0.31,1.65)	0.98 (0.39,2.52)
	Yes	73 (25.89)	209 (74.11)	1.00	1.00
Knowledge of PP cxn.	No	63 (32.14)	133 (67.86)	**0.36 (0.21,0.61)**	0.48 (0.26,0.90)*
	Yes	24 (14.55)	141 (85.45)	1.00	1.00

* Statistically significant associations *p* < 0.05, *cxn* Complication, *COR* Crude odds ratio, *AOR* Adjusted odds ratio

## Discussion

According to this study more than 75% of pregnant women were not well prepared for childbirth and its complications. The finding was low in comparison to the WHO standard which states every pregnant woman should have a birth plan [16]. However, it’s relatively higher than findings of studies conducted in some parts of Ethiopia- (Sidama Zone (17%) [10], Arsi Zone (16.5%) [11], and Wolita Zone (18.3%) [17]). But the result was nearly similar with that of South Wollo (24.1%) [18], Adigrat of Tigray Region (22%) [9], Basoliben in Amhara Region (26.9%) [19]. However, it was far-less than findings from the studies performed in major cities of Ethiopia such as Dire Dawa, (54.7%) [20] and Addis Ababa, (56.3%) [21], and findings from other countries like India (47.8%) [22] and Nigeria (82.1%) [23], those variations could happen due to the difference in the study settings, socio-cultural characteristics, political-economic features, and implementation of related health programs.

We know that learning has multidimensional influences on human life practice and behavior. In this study women who had no formal education (AOR = 0.36, 95%; CI (0.15,0.85)) and those who can only read and write (AOR = 0.41, 95%; CI (0.19,0.89)) were less likely to be well prepared compared to those who had secondary level education. The finding was analogous to other studies conducted in different parts of Ethiopia and other countries [7, 10, 13, 22–25]. Educated women have more opportunities and a tendency for BP/CR as education increases the acquisition of information. Because the majority of women (64%) had no formal education [Table 1], an improvement of formal education at a young age could have the greatest maternal health benefit. As the current study setting is very remote (i.e 400-k meters away from Semera, the capital city of the Afar Regional State) with the less developed infrastructure it needs to improve access to health service and education.

Women who do not know BP/CR [AOR = 0.48; CI (0.26, 0.90)] were found to have fewer odds of being well prepared for childbirth and readiness for complications compared to those women who know. The result indicates more than 80% of the women reported [Table 3] they had the information (i.e awareness). However, the source of information was not the same. Of those who had information (42/319), 60.11% received it from health professionals, (44/319) 12.19% from Health Extension Workers [Table 3]. It seems the difference between health professionals and Health Workers as a source of information is wide. The gap still is seen between health education provision on complication readiness or knowledge about it like foul-smelling vaginal discharge, (47/361), [13.02%], and convulsion [4.43%], high-grade fever, (41/361), [11.36%], [Table 4], compared to information on BP/CR, (319/361), (88.37%), [Table 3]. This shows there is a need to improve ways of providing complete information when counseling about BP/CR.

Providing income generation and socioeconomic opportunities for women will have a good impact on BP/CR. The women/families who had less average monthly income were found to have less BP/CR scores. On the contrary, compared to women who have monthly income of more than 2000 ETB, those with a monthly income of less than 1000 (AOR = 0.21, 95%; CI (0.07, 0.67)), and 1000 to 2000 (AOR = 0.38, 95%; CI (0.19, 0.76), were less likely to have BP/CR (Table 5). Similar findings were reported in other studies [11, 15, 25]. Hence, a better economic status might strengthen BP/CR. Besides, women who were not aware of postpartum danger signs had less BP/CR (AOR = 0.48, 95%; CI (0.26, 0.90)). The finding was similar to other studies conducted in Ethiopia and Rural Uganda [7, 11, 26].

Maternity issues include a different spectrum of life about risks and successes. The logistic regression analysis for the current data revealed that those women of younger age groups were less prepared to birth and related complications [18–19, (AOR = 0.18, 95%; CI (0.04, 0.94))], and [20–34, (AOR = 0.40, 95% CI (0.20, 0.78))] when compared to (35–49. This was similar to findings from a study in Nigeria [23].

There is no argument that religion could have either a positive or negative influence on the use of health services. This study revealed Muslim women were less likely to be well prepared for birth and related complications (AOR =0.40, 95% CI (0.18, 0.85)) compare to Protestants. This may be related to the effect of religion on culture and the preferred method of delivery. However, further qualitative studies are required more to explore relations.

This study tried to minimize selection bias by employing by giving all eligible women in the community an equal chance to be selected. The limitation is that associations do not demonstrate the cause and effect relationships as this is a cross-sectional study.

Besides, since this study focused on one district the results might not be conclusive to the region at large.

## Conclusions

The prevalence of BP/CR in the study area was far less than the World Health Organization requirement. Women’s educational status, average monthly family income, and women’s knowledge of postpartum obstetric complications were independent predictors of BP/CR. Governmental and non-governmental organizations should strive to improve women’s education, income, and awareness of birth-related complications.

## Supplementary information

**Additional file 1.**

## Data Availability

The authors confirm the availability of the data and can be accessed on ethically reasonable request though the analyzed data is in the manuscript.

## Abbreviations

BP/CR: Birth preparedness and complication readiness
CSAE: Central Statistical Agency [Ethiopia]
EDHS: Ethiopian Demographic and Health Survey
EmOC: Emergency Obstetric Care
HDSS: Health and Demographic Surveillance System
IAG: Inter-Agency Group
SMI: Safe Motherhood Initiative
TBA: Traditional Birth Attendants
TTBA: Trained Traditional Birth Attendants
UNFPA: United Nation Fund Population Activity
UNICEF: United Nations International Children’s Emergency Fund
WHO: World Health Organization
