# Supplementary material for: Birth preparedness complication readiness and determinants among pregnant women: a community-based survey from Ethiopia
Source: BMC Pregnancy Childbirth. 2020 Oct 19;20:631. doi: 10.1186/s12884-020-03297-w (PMC7574236; doi:10.1186/s12884-020-03297-w)
Supplement: Supplementary file 1 — Additional file 1. [file 12884_2020_3297_MOESM1_ESM.docx]

# ANNEX A: ENGLISH VERSION QUESTIONER

Interviewer visits

| Number of Visits | | | |
| --- | --- | --- | --- |
|  | 1 | 2 | 3 |
| Interviewer name |  |  |  |
| Date |  |  |  |
| Result |  |  |  |

Result codes:

**1 =** Completed, **2 =** Women absent (specify the reason),**3 =** Time and date set for later,**4 =** Incomplete interview, **5 =** Refused and **6 =** other (specify) ----------------------

**Identification Information**

001. Code No. ------------------

002. Kebele--------------------- Sub-kebele(Gote )---------------------------- House no.-----------

| Section 1: Socio demographic Information | | | | | | | |
| --- | --- | --- | --- | --- | --- | --- | --- |
| Q.# | **Question** | **Codes** | | | | | **Go to Q.#** |
| 101 | Location of kebele | Rural…………………….1  Urban……………………2 | | | | |  |
| 102 | Age in completed years | _________________ | | | | |  |
| 103 | Marital status | Single-------------------------------------1  Married/in union …………............2  Widowed ...……………….............3  Divorced/separated .……………..4 | | | | |  |
| 104 | Religion | Orthodox ………………........1  Catholic ………………..........2  Protestant ……………….......3  Muslim ………………….........4  Other ………………………...99  (Specify)…………………….. | | | | |  |
| 105 | Ethnicity | Afar …. …………………....... 1  Amhara …………………....... 2  Oromo…………………..……..3  Tigray………………….………4  SNNP ………………………….5  Other ………………..99 (Specify)……………… | | | | |  |
| 106 | Occupation | Cattel production …………… 1  Govt. employee …………...... 2  Private employee ………....... 3  Private business.………......... 4  Other ……………….99(Specify)……………… | | | | |  |
| 107 | Educational status | Not read & write …………....... 1  Read & write ………………..... 2  Primary………………………….3  Secondary & above----------------4 | | | | |  |
| 108 | House hold Monthly income in Ethiopian Birr | ------------------------------------------ | | | | |  |
| 109 | Family size | --------------------------- (in number) | | | | |  |
| Question no.110 – 111 will be asked if the answer for question no.103 is married/in union. | | | | | | | |
| 110 | Husband’s occupation | Cattel production ……………1  Farmer……………………….2  Govt. employee …………......3  Private employee ………....... 4  Private business …………..... 5  Other ……….………99 (Specify)……………… | | | |  | |
| 111 | Husband’s educational status | Not read & write …………....... 1  Read & write ………………..... 2  Primary………………………….3  Secondary & above ...……….. 4 | | | |  | |
| Section 2: Obstetrics factors | | | | | | | |
| Q.# | **Question** | | | **Codes** | | **Go to Q.#** | |
| 201 | How many months pregnant are you? | | | < 3 months………………1  3-6months…………….2  >7months…………….3 | |  | |
| 202 | Is this your first pregnancy? | | | Yes……………………1  No…………………….2 | | **If yes Go to Q#.204** | |
| 203 | How many times in total you  became pregnant? | | | ------------------( in number) | |  | |
| 204 | Did you attend antenatal care during this pregnancy? | | | Yes……………………1  No…………….……….2 | | If No Go to Q.# **207** | |
| 205 | How many times did you attend antenatal care during this pregnancy? | | | Only one………………….1  Two times……..………….2  Three times…….…………3  Four and above…………..4 | |  | |
| 206 | Whom did you first see for check upon this pregnancy? | | | Health professional ….1  HEW…………………..2  Do not know…….…………3  Other .(specify…………..99 | |  | |
| 207 | How many times in total you gave birth? | | | -------------------( in number) | |  | |
| 208 | How many of your pregnancies  resulted in a baby that was born alive? | | | ------------------- (in number) | |  | |
| 209 | Have any of your pregnancies resulted in a baby that was born dead (a stillbirth)? | | | Yes………………………1  No………………….2 | | If No Go to **Section 3** | |
| 210 | If yes how many? | | | ------------------- (in number) | |  | |
| Section 3:Knowledge of Danger sign | | | | | | | |
| Q.# | **Question** | | **Codes** | | | **Go to Q.#** | |
| 301 | Do you know any/some serious health problem/s that can occur **during pregnancy** that could endanger the life of a pregnant woman? | | Yes ……………………… 01  No …………….................02 | | | If No Go to Q.# **303** | |
| 302 | Can you mention them?  (Probe: any others?) | | 1.Vaginal bleeding ………...01  2.Blurred vision………….…02  3.Swollen hands/face ……...03  4.Convulsions………….…...04  5.Loss of consciousness …….…05  6.Severe abdominal pain…….....06  7.Accelerated/reduced fetal movement ……………………07  8.Water breaks without labor………………………..….08  9.Other (Specify………..………99 | | | Circle for  spontaneous  answers. | |
| 303 | Do you know any/some serious health problem/s that can occur during labor and childbirth that  could endanger the life of a pregnant woman? | | Yes ……………………… 01  No ………….................... 02 | | | If No Go to Q.#  **305** | |
| 304 | Can you mention them?  (Probe: any others?) | | 1.Severe vaginal bleeding……..01  2.Convulsions…………..………02  3.Loss of consciousness.……..03  4.Labor lasting >12 hour……....04  5.Placenta not delivered 30 minutes after baby………………05  6.Other. (Specify)……………….99 | | | Circle for  spontaneous  answers. | |
| 305 | Do you know any/some serious health problem/s that can occur during the first 42 days after birth that could endanger the life of the woman? | | Yes ……………………… 01  No …………………......... 02 | | | If No Go to Q.# **307** | |
| 306 | Can you mention them?  (Probe: any others?) | | Severe vaginal bleeding………..01  High fever …………………..02  Loss of consciousness…………03  Foul- smelling vaginal discharge………………………04  Other. (specify…………………..99 | | | Circle for  spontaneous  answers. | |
| 307 | Have you ever heard the term“birth preparedness and complication readiness’’? | | Yes ………………………… 01  No …………………...............02 | | | If No Go to Q.#  **309** | |
| 308 | From whom did you get the information? | | Health professional ……………01  TTBA ………………………….02  TBA ……………………………03  HEW…………………………..04  Mothers ……………………….05  Media…………………………..06  Other.(specify…………………..99 | | | Circle for  spontaneous  answers. | |
| 309 | In your opinion, what are some things a woman can do to prepare for birth?  (Probe: which of the following do you think a preparation for birth?) | | Identify place of delivery and Identifying the nearest institution that has 24 hour functioning EmOC services………………….01  Save money for emergency…...02  Identify skilled provider……...03  Arranging blood donors…...04  Identify a mode of transportation during obstetric emergency……05  Other . (specify…………….99 | | | Circle for  spontaneous  answers. | |
| Section 4: Practices of respondents on preparation for birth. | | | | | | | |
| Q.# | **Question** | | | | **Codes** | **Go to Q.** | |
| 401 | Do you Identify place of delivery? | | | | Yes-------------------------01  No--------------------------02 | **If No Go to Q.# 403** | |
| 402 | If the response for Q. #401 is yes Where do you plan to give birth to this baby? | | | | Home……………….01  Health Institutions…02 |  | |
| 403 | Who do you expect will assist you with the birth?  PROBE FOR THE TYPE OF PERSONAND RECORD ALL PERSONS  MENTIONED. | | | | Health professional ….01  TTBA ……………….02  TBA …...……………03  HEW ………………..04  Mothers ………….…05  Do not know..……..06  Others ………………...99  Specify---------------------- |  | |
| 404 | Are you saving money for the purpose of pregnancy and child birth? | | | | Yes-------------------------01  No--------------------------02 |  | |
| 405 | Are you preparing essential items for clean delivery & post partum? | | | | Yes-------------------------01  No--------------------------02 |  | |
| 406 | Have you decided to deliver in health institutions by skilled provider? | | | | Yes-------------------------01  No--------------------------02 |  | |
| 407 | Have you Identified mode of transportation during pregnancy and delivery time? | | | | Yes-------------------------01  No--------------------------02 |  | |
| 408 | How did you go to the health facility? | | | | Ambulance..................... 01  car.................................. 02  Cart................................ 03  On foot ......................... 04  Hores/Donkey-----------05  Don’t know…. ....... .....06  Other ____________ 99  (SPECIFY) |  | |
| 409 | Have you arranged blood donor for complication faced in relation to pregnancy and delivery? | | | | Yes------------------------01  No------------------------02 |  | |
| 410 | Have you Identified institution with 24 hrEmOC services? | | | | Yes------------------------01  No-------------------------02 |  | |

**This is the end of the interview.**

**THANK YOU!**

Date of data collection………………………………………..

Name of data collector……………………………………….

Signature……………………………………………………..

Name of supervisor………………………………………….

Signature……………………………………………………

Date----------------------------------------

# ANNEX C: AMHARIC VERSION QUESTIONER

**የቃለመጠይቅ አድራጊው ጉብኝት**

**የውጤት ኮዶች:**

**1 =**መጠይቁ ተሞልቷል **2 =**በጉብኝት ሰዓት አልተገኘችም **3 =**መጠይቁ ለሌላ ጊዜና ሰዓት ቀጠሮ ተያዘለት **4 =**ሙሉ በሙሉ ያልተሞላ መጠይቅ **5 =**ለቃለ መጠይቁ ፍቃደኛ አልሆነችም **6 =**ሌላ ምክንያት( ይጠቀስ) -------

**መለያመረጃ**

001. የኮድቁጥር፡ -----------------------

002. ቀበሌ፡---------------------------------------ጎጥ---------------------------------------የቤ.ቁ፡ ---------------------------------

| ክፍል 1፡ስነህዝብናማህበራዊ ጉዳዮች | | | | | | | |
| --- | --- | --- | --- | --- | --- | --- | --- |
| ጥያቄ ቁ. | **ጥያቄ** | **ኮዶች** | | | | | **ወደ ጥያቄ ቁጥር --------እለፍ/ፊ** |
| 101 | የቀበሌው ዓይነት | ገጠር…………………….1  ከተማ……………………2 | | | | |  |
| 102 | እድሜ (በሙሉ ዓመት ይሞላ) | _________________ | | | | |  |
| 103 | የጋብቻ ሁኔታ | ያላገቡ --------------------------------------------1  ያገቡ /አብረው የሚኖሩ-------------------------2  ባልየሞተባቸው----------------------------------3  የፈቱ----------------------------------------------4 | | | | |  |
| 104 | ሐይማኖት | ኦርቶዶክስ--------------------1  ካቶሊክ-----------------------2  ፕሮስቴስታንት---------------3  ሙስሊም--------------------4  ሌላ-------------------------99 (ይጠቀስ) ……………… | | | | |  |
| 105 | ብሔር | አፋር-------------------------1  አማራ …………………………………. 2  ኦሮሞ ------------------------3  ትግሬ------------------------….4  ሌላ -------------------------99 ( ይጠቀስ)----------------------- | | | | |  |
| 106 | ስራ | አርብቶ አደር..-----------------------------1  ግብርና-------------------------------------2  የቤት እመቤት-----------------------------3  የመንግስት ሰራተኛ------------------------4  የግልተቀጣሪ------------------------------5  የግል ንግድ ሥራ------------------------- 6  ሌላ---------------------------------------99  (ይጠቀስ)-------------------------------------- | | | | |  |
| 107 | የትምህርት ሁኔታ | መፃፍ ማንበብ የማትችል------------------1  መፃፍ ማንበብ የምትችል------------------2  አነደኛ ደረጃ-------------------------------3  ሁለተኛ ደረጃ እና ከዚያ በላይ------------4 | | | | |  |
| 108 | ወርሃዊ የቤተሰብ ገቢ (በብር) | ---------------------------------------- | | | | |  |
| 109 | የቤተሰብ ብዛት | --------------------------- (በቁጥር) | | | | |  |
| ከጥያቄ ቁጥር 110-111 ያሉት ጥያቄዎች የሚጠየቁት የጥያቄ ቁጥር 103 መልስ ያገቡ/ አብረው የሚኖሩ ከሆነ ብቻ ነው፡፡ | | | | | | | |
| 110 | የባል ስራ | | አርብቶ አደር..-----------------------------1  ግብርና-------------------------------------2  የመንግስት ሰራተኛ------------------------.3  የግል ተቀጣሪ------------------------------4  የግልንግድ ሥራ------------------------- 5  ሌላ---------------------------------------99  (ይጠቀስ)-------------------------------------- | | | |  |
| 111 | የባል የትምህርት ሁኔታ | | መፃፍ ማንበብ የማትችል------------------1  መፃፍ ማንበብ የምትችል------------------2  አነደኛ ደረጃ-------------------------------3  ሁለተኛ ደረጃ እና ከዚያ በላይ------------4 | | | |  |
| ክፍል 2. ፡የዕርግዝናእናየወሊድሁኔታ | | | | | | | |
| ጥያቄ ቁ. | **ጥያቄ** | | | | **ኮዶች** | | **ወደ ጥያቄ ቁጥር ---- እለፍ/ፊ** |
| 201 | እርግዝናው በወር ሲሰላ ምን ያህል ግዜ ይሆነዋል? | | | | ከ 3 ወር በታች………………1  ከ3 አስከ 6 ወር….……………….2  ከ7 ወር በላይ.…….…………….3 | |  |
| 202 | ይህ እርግዝና የመጀመሪያሽ ነው? | | | | አዎ…………………………1  አይደለም…………………….2 | | **መልሱ አዎ ከሆነ ወደ ጥያቄ ቁጥር204 እለፍ/ፊ** |
| 203 | በአጠቃላይስንተኛ እርግዝናዎ ነው? | | | | ----------------------(በቁጥር) | |  |
| 204 | በአሁኑ እርግዝና የቅድመ ወሊድ የጤና ምርመራ አድርገው ያውቃሉ? | | | | አዎ…………………………1  አላደረኩም.…….…………….2 | | **መልሱ አላደረኩም ከሆነ ወደ ጥያቄ ቁጥር207እለፍ/ፊ** |
| 205 | በአጠቃላይ ምን ያህል ግዜ የቅድመ ወሊድ ምርመራ አድርገዋል? | | | | አንድ ግዜ.………………….1  ሁለት ግዜ……..…..……….2  ሶስት ግዜ .……...…….……3  አራትና ከዚያ በላይ…………..4 | |  |
| 206 | በዚህ የእርግዝና ወቅት ለመጀመሪ ግዜ ምርመራ ደረገልዎት ማን ነበር? | | | | የጤና ባለሙያ ……………...….1  የጤና ኤክስቴንሽን ባለሙያ…..…..2  አላውቀውም….………….……3  ሌላ ካለ የጠቀስ………..……..99 | |  |
| 207 | በአጠቃላይ ምን ያህል ጊዜ ልጅ ወልደዋል? | | | | ---------------------- | |  |
| 208 | ከእርግዝና ጊዜዎት ውስጥ ምን ያህል ጊዜያት ህይወት ያለው ልጅ ወልደዋል? | | | | --------------------- | |  |
| 209 | ከዚህ በፊት በነበርዎት እርግዝና ህይወቱ ያለፈ ልጅ ወልደው ያውቃሉ? | | | | አዎ…………………………1  አይ………………………….2 | | **መልሱ አይ ከሆነ ወደ ክፍል 3እለፍ/ፊ** |
| 210 | ምን ያህል ጊዜያት ህይወቱ ያለፈ ልጅ ወልደዋል? | | | | --------------------- | |  |
| ክፍል 3 ፡ እውቀት | | | | | | | |
| ጥያቄ ቁ. | **ጥያቄ** | | | **ኮዶች** | | | **ወደ ጥያቄ ቁጥር---- እለፍ/ፊ** |
| 301 | በእርግዝና ወቅት ህይወትን አደጋ ላይ ይጥላል ወይም ይጥላሉ የሚባሉ ዋና ዋና የጤና ችግሮች ያውቃሉ? | | | አዎ ……………………… 1  አይ……............................2 | | | **መልሱ አይ ከሆነ ወደ ጥያቄ ቁጥር303 እለፍ/ፊ** |
| 302 | መልስዎ አዎ ከሆነ የጤና ችግሮችን መጥቀስ ይችላሉን?  (ሌላስ?) | | | 1. የሴት ብልት መድማት------------------- 1  2. የእይታ ችግር-----------------------------2  3. የፊት/የእጅ እብጠት--------------------- 3  4. መንቀጥቀጥ------------------------------4  5. እራስን መሳት-----------------------------5  6. ከፍተኛ የሆድ ህመም----------------------6  7. የተፋጠነ/ወይም የዘገመ የሽል እንቅስቃሴ..-7  8. ምጥ በሌለበት የእንሽርት ውሃ መፍሰስ--8  9. ሌላካለይጥቀሱ----------------------99 | | | የተጠቀሱ መልሶችን አክብብ/ቢ. |
| 303 | በምጥና በልጅ መውለጃ ጊዜ ሊከሰቱ የሚችሉ እናትን አደጋ ላይሊጥሉ የሚችሉ ዋና ዋና የጤና ችግሮች ያውቃሉ? | | | አዎ ……………………….……… 01  አይ …………............................. 02 | | | **መልሱ አይ ከሆነ ወደ ጥያቄ ቁጥር305 እለፍ/ፊ** |
| 304 | መልስዎ አዎ ከሆነ የጤና ችግሮችን መጥቀስ ይችላሉን?  (ሌላስ? በማለት እንዲናገሩ መጋበዝ | | | 1. ከፍተኛ የሴት ብልት መድማት------------ 1  2. መንቀጥቀጥ---------------------------------2  3. እራስን መሳት-------------------------------3  4. ከ12 ሰዓት በላይ የሚቆይ ምጥ--------------4  5.የእንግዴ ልጅ ህፃኑ ከተወለደ ከ30 ደቂቃ በላይ ከቆዬ-----------------------------------5  6. ሌላካለይጥቀሱ----------------------99 | | | የተጠቀሱ መልሶችን አክብብ/ቢ. |
| 305 | የወለደችን ሴት ህይወት አደጋ ላይ ሊጥል የሚችሉ እና ከወሊዱ በኋላ በመጀመሪያዎቹ 42 ቀናት ውስጥ ሊከሰቱ ሚችሉ ማንኛውም ወይም በጤና ላይ ከፍተኛ ችግር የሚያስከትሉ ነገሮችን ያውቃሉ? | | | አዎ ……………………….……… 01  አይ …………............................. 02 | | | **መልሱ አይ ከሆነ ወደ ጥያቄ ቁጥር307 እለፍ/ፊ** |
| 306 | መልስዎ አዎ ከሆነ የጤና ችግሮችን መጥቀስ ይችላሉን?  (ሌላስ? በማለት እንዲናገሩ መጋበዝ | | | 1. ከፍተኛ የሴትብልት መድማት------------ 1  2. ከፍተኛ ትኩሳት----------------------------2  3. እራስን መሳት-------------------------------3  4. ሽታ ያለው ከሴት ብልት የሚወጣ ፈሳሽ-----4  5. ሌላ ካለ ይጥቀሱ----------------------99 | | | የተጠቀሱ መልሶችን አክብብ/ቢ. |
| 307 | የወሊድ ዝግጁነትና ሊያጋጥምየሚችሉችግሮችተዘጋጅቶስለመጠበቅየሚባል መረጃ ሰምተው ያውቃሉ? | | | አዎ ……………………………… 01  አይ ………………….................... 02 | | | **መልሱ አይ ከሆነ ወደ ጥያቄ ቁጥር309 እለፍ/ፊ** |
| 308 | መልስዎ አዎን ከሆነ መረጃን ከማን አግኝተዋል? | | | ከጤና ባለሙያ……………………….01  ከሰለጠነ የልምድ አዋላጅ……………….02  ከልምድ አዋላጅ………………………03  ከጤና ኤክስቴንሽን ባለሙያ……………..04  ከእናቶች …………………………….05  ከመገናኛ ብዙሃን………….…………..06  ሌላ ካላ ይጠቀስ………………..……..99 | | | የተጠቀሱ መልሶችን አክብብ/ቢ. |
| 309 | በእርስዎ አስተያየት ሴቶች ለወሊድ ሲዘጋጁ የሚያደርጉት መሰናዶ ምንድንነው? | | | 1. የምትወልድበት ቦታን ለይቶ ማወቅና እንዲሁም በቅርብ ያለው እና የ24 ሰአት የድንገተኛ ሕክምና አገልግሎት የሚሰጥ ተቋምን ለይቶ ማወቅ-------------------------------------1  2.በእርግዝናና በወሊድ ወቅት አገልግሎት ላይ ሚውል ገንዘብ ማጠራቀም----------------------2  3. የሰለጠነ አዋላጅ የጤና ባለሙያ ለይቶ ማወቅ--------------------------------------------3  4. ደም ለጋሾችን ማዘጋጀት----------------------4  5. በአደጋ ጊዜ የትራንስፖርት አይነት ለይቶ ማወቅ--------------------------------------------5  ሌላ (ይግለፁት) -----------------------------99 | | | የተጠቀሱ መልሶችን አክብብ/ቢ. |
| ክፍል 4፡ ለወሊድ ያለውን ዝግጅት አስመልከቶ የመልስ ሰጪዎች ተግባር | | | | | | | |
| ጥያቄ ቁ. | **ጥያቄ** | | | | | **ኮዶች** | **ወደ ጥያቄ ቁጥር ---- እለፍ/ፊ** |
| 401 | የወሊድ ቦታን ለይተው አውቀዋልን? | | | | | አዎ----------------------------01  አይ ---------------------------02 | **መልሱ አይ ከሆነ ወደ ጥያቄ ቁጥር403 እለፍ/ፊ** |
| 402 | የወሊድ ቦታን ለይተው ካወቁ የት ለመውለድ ነው ያቀዱት? | | | | | መኖሪያ ቤት……..………….01  የጤና ተቋም…………………02 |  |
| 403 | ይህንን እረግዝና ማን ያገላግለኛል/ያዋልደኛል ብለው ያስባሉ? | | | | | የጤና ባለሙያ ……………….01  የሰለጠነ የልምድ አዋላጅ……….02  የልምድ አዋላጅ………………03  የጤና ኤክስቴንሽ ባለሙያ……..04  እናቴ …………………….…05  አላውቅም……………….…..06  ሌላ ካለ ይጠቀስ……………...99 |  |
| 404 | በእርግዝናና በወሊድ ሰዓት የሚሆን የአስቸኳይ ጊዜ ገንዘብ አዘጋጅተዋል? | | | | | አዎ----------------------------01  አይ-----------------------------02 |  |
| 405 | ለንጹህ የወሊድ አገልግሎት እና ከወሊድ በኋላ ላሉት ጉዳዮች አስፈላጊ ነገሮችን እያዘጋጁ ነው? | | | | | አዎ----------------------------01  አይ-----------------------------02 |  |
| 406 | በጤና ተቋም ውስጥ እና በሰለጠነ የጤና ባለሙያ ለመውለድ ወስነዋልን? | | | | | አዎ----------------------------01  አይ-----------------------------02 |  |
| 407 | በእርግዝናና በወሊድ ወቅት ወደ ጤና ተቋም የሚሄዱበት የትራንስፖርት/መጓጓዣ አይነትን ለይተው አውቀዋልን? | | | | | አዎ------------------------------01  አይ-----------------------------02 |  |
| 408 | ወደ ጤና ተቋም የሚሄዱበት ትራንስፖርት/መጓጓዣ ምን ዓይነት ነው ? | | | | | በአንቡላንስ-----------------------01  በመኪና---------------------------02  በጋሪ------------------------------03  በእግር----------------------------04  በሰው ሸክም ---------------------05  አላውቅም-------------------------06  ሌላ ካለ ይጠቀስ------------------99 |  |
| 409 | በእርግዝናና በወሊድ ሰዓት ለሚገጥሙ የጤና ችግሮች ደም ለጋሾችን አዘጋጅተዋልን? | | | | | አዎ------------------------------01  አይ-----------------------------02 |  |
| 410 | የ 24 ሰዓት የድንገተኛ ሕክምና አገልግሎት የሚሰጥ ተቋምን ለይተው አውቀዋልን? | | | | | አዎ------------------------------01  አይ-----------------------------02 |  |

**መጠይቁን ጨርሻለሁ አመሰግናለሁ!**

መጠይቁ የተሞላበት ቀን………………………መጠይቁን የሞላው ስም………………………………

ፊርማ……………………………………………………..

የሱፐርቫይዘር ስም………………………………………….ፊርማ………………………………

ቀን …………………………………………………….
